# Alternative splicing of HSPA12A pre‐RNA by SRSF11 contributes to metastasis potential of colorectal cancer

**DOI:** 10.1002/ctm2.1113

**Published:** 2022-11-17

**Authors:** Yao‐Jie Pan, Fu‐Chun Huo, Meng‐Jie Kang, Bo‐Wen Liu, Meng‐Di Wu, Dong‐Sheng Pei

**Affiliations:** ^1^ Laboratory of Clinical and Experimental Pathology Xuzhou Medical University Xuzhou China; ^2^ Department of General Surgery Xuzhou Medical University Xuzhou China

**Keywords:** alternative splicing, colorectal cancer, EMT, metastasis, phosphorylation, ubiquitination

## Abstract

**Background:**

Dysregulation of alternative splicing (AS) induced by serine/arginine‐rich proteins has recently been linked to cancer metastasis. Nonetheless, as a member of the serine/arginine‐rich protein family, the involvement of SRSF11 in colorectal cancer (CRC) is unknown.

**Methods:**

The TCGA dataset and clinical samples were used to assess SRSF11 expression levels in CRC. For SRSF11, functional experiments were conducted both in vitro and in vivo. RNA‐seq technology was used to analyze and screen SRSF11‐triggered AS events, which were then confirmed by in vivo UV crosslinking and immunoprecipitation (CLIP) and mini‐gene reporter assays. Jalview software was used to determine the preferential binding motif with relation to exon skipping (ES) events. Furthermore, coimmunoprecipitation (Co‐IP) and Phospho‐tag SDS‐PAGE experiments were used to investigate PAK5‐mediated phosphorylation regulation on SRSF11, and in vitro kinase experiments validated the interaction.

**Results:**

In CRC, SRSF11 was discovered to be overexpressed and associated with a poor prognosis. And SRSF11 played a pro‐metastatic role in vitro and in vivo. By screening SRSF11‐regulated AS events, we identified the binding motif of SRSF11‐triggered splicing‐switching of HSPA12A AS, which specifically regulated HSPA12A AS by directly binding to a motif in exon 2. Mechanistically, the HSPA12A transcript with exon 2 retention increased N‐cadherin expression by promoting RNA stability. Furthermore, the oncogenic kinase PAK5 phosphorylated SRSF11 at serine 287, protecting it from ubiquitination degradation.

**Conclusions:**

SRSF11 exerts pro‐metastatic effects in CRC by inhibiting the AS of HSPA12A pre‐RNA. Our findings point to SRSF11‐regulated HSPA12A splicing as a novel relationship between SRSF11‐regulated splicing and CRC metastasis and suggest a PAK5/SRSF11/HSPA12A axis as a potential therapeutic target and prognostic biomarker in CRC.

## Graphical abstract

- PAK5 facilitates the nuclear translocation and alternative splicing function of SRSF11 on HSPA12A through its phosphorylation regulation.
- SRSF11 inhibits HSPA12A exon 2 skipping, which benefits CRC metastasis.
- Exon 2‐inclusive HSPA12A transcripts increase the stability of N‐cadherin mRNA.

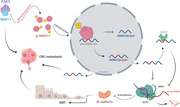

## 1 INTRODUCTION

With a continually high prevalence, particularly in those under the age of 50, colorectal cancer (CRC) has risen to become the second most common cancer fatality globally.^1^ Approximately 20% of newly diagnosed CRC patients suffer from distant metastasis, and 25%–50% advance to remote metastasis within 3 years.^2^ Although the primary tumour could be excised by radical resection, almost 50% of patients developed postoperative metastases, and less than 10% of patients survived for 5 years without adequate treatment.^3^ The overall survival (OS) rate of advanced CRC has improved as a result of extensive chemoradiotherapy.^4^ However, efficacy remains low in patients with distant metastases. Therefore, exploring metastasis‐associated genes and research into the regulatory role of the mechanism can reveal prospective targets and ideas for CRC treatment, which is critical for enhancing clinical efficacy and OS rates.

Alternative splicing (AS) occurs at the transcriptional level in up to 95% of human genes, increasing the richness and diversity of transcripts and protein isoforms.^5^ Exon skipping (ES), intron retention, alternative 5′ splice site, alternative 3′ splice site and mutually exclusive exon are five basic categories that define the patterns of AS events. In general, the most common is ES. Furthermore, abnormal AS events have been discovered and associated with the majority of human diseases, including cancer.^6^ Aberrant AS‐triggered splice‐switching of transcripts or protein isoforms plays a significant role in cancer progression, impacting characteristics such as cell proliferation and apoptosis, cell cycle, metastasis, angiogenesis, metabolism and immunological evasion. Although other genes, including ZO‐1, PKM, RBFOX2 and PTBP1, have been reported to have aberrant AS in CRC, the fundamental link connecting splicing factors (SFs) and alternative transcripts or isoforms remains elusive.^7^, ^8^, ^9^

A multicomponent complex known as a spliceosome is the primary regulator of the AS process. It is made up of five small nuclear ribonucleic acids (snRNAs) and several hundred protein components. Because of the interaction between SFs and the spliceosome, the AS processes completed the steps of recognition, assembly, catalysis and dissociation.^10^ Subordinating to the SF family, all of the serine/arginine‐rich proteins possess at least one RNA recognition motif (RRM) and one arginine/serine‐rich (RS) domain.^11^ Serine/arginine‐rich proteins, in particular, use the RRM domain to locate and combine with splicing regulatory elements (SREs), but the RS domain functions as a signal for nuclear localization and spliceosome formation.^12^

Notably, due to the abundance of arginine and serine sequences in the RS domain, posttranslational modifications such as phosphorylation prefer it.^13^, ^14^ The protein kinase families SRPK, CLK, topoisomerase 1, PRPF4B, NEK2 and AKT are six identified families that can phosphorylate the RS domains and potentially change the cellular distribution and splicing process of serine/arginine‐rich proteins. It is worth noting that serine/arginine‐rich proteins may be over‐active just by hyperphosphorylation, not necessarily an increase in expression. The oncogenic significance of p21‐activated kinase 5 (PAK5) in CRC has sparked widespread attention in basic and clinical cancer research,^15^, ^16^, ^17^, ^18^ despite the fact that its function in phosphorylation‐related regulation of serine/arginine‐rich proteins remains unknown.^14^, ^19^

Changes in the expression levels of general or specific SFs are one of the primary causes of abnormal AS processes in cancer, which leads to the synthesis of previously unknown RNAs or changes in the ratios between existing isoforms, with consequent implications on oncogenesis and tumour growth.^20^ As a result, serine/arginine‐rich proteins alter pre‐RNA splicing, influencing carcinogenesis. For example, SRSF1 regulates lung cancer radioresistance by modulating PTPMT1 splice‐switching.^21^ SRSF6 also regulates the AS of ZO1 pre‐RNA, which promotes tumour development in CRC.^7^ Other biological activities of serine/arginine‐rich proteins include mediating nuclear export of mRNA, regulating RNA stability, and modulating translation; however, regulating AS is the most important.^11^ As a key serine/arginine‐rich protein, SRSF11 has been shown to block ES of target pre‐RNAs such as F1γ and neuronal microexons while promoting ES of tau, SMN2 and ZNF207 pre‐RNAs.^22^, ^23^, ^24^, ^25^, ^26^ Nonetheless, SRSF11 is understudied and its role in carcinogenesis, particularly CRC, is uncertain.

We sought to explore the expression levels of SRSF11, as well as its clinical correlation, biological function, and underlying AS‐associated function of CRC. SRSF11 was discovered as an elevated gene and an indicator of poor prognosis in analyses of the TCGA database and clinical samples. RNA‐seq technology was used to explore SRSF11‐triggered AS events, which were then validated by in vivo CLIP and mini‐gene reporter assays, revealing a preferential binding motif for ES events. Then, we proved that SRSF11 played a pro‐metastatic role in CRC cells by modulating HSPA12A splicing, which led to N‐cadherin mRNA stability protection. Furthermore, we uncovered PAK5‐regulated phosphorylation, which resulted in the protein stability of oncogenic SRSF11. Our findings shed light on the role of SRSF11 in the progression of CRC metastasis and elucidate the underlying mechanisms in detail.

## 2 MATERIALS AND METHODS

### 2.1 Cell lines, reagents, lentivirus generation and stable cell line construction

LoVo, HCT116, SW480, SW620, DLD‐1, HT‐29 and FHC cell lines were obtained from the General Surgery Department of Xuzhou Medical University. Cells were cultured in DMEM (Bio‐Channel, catalog: BC‐M‐005) or RPMI 1640 (Bio‐Channel, catalog: BC‐M‐017) containing 10% FBS (ExCell Bio, catalog: FSP500). The incubation conditions of cells are 5% CO_2_, at 37°C. Actinomycin D (ActD) (MCE, catalog: HY‐17559), MG132 (MCE, catalog: HY‐13259) and cycloheximide (CHX) (MCE, catalog: HY‐12320) were used to inhibit transcription, proteasome and protein synthesis, respectively. Alkaline phosphatase (AP) (ThermoFisher, catalog: EF0651) was used as a mediator reversing phosphorylated protein into unphosphorylated status.

A suite of SRSF11 siRNAs was synthesized by GenePharma, and si‐HSPA12A was synthesized by IBSBIO followed by transfection using jetPRIME (Polyplus Transfection, catalog: EF0651) based on the manufacturer's instructions. The sequence of SRSF11 siRNAs are as follows: SRSF11^#1^ Sense: GGAUACCUCUAGUAAAGAAAU, Anti‐Sense: UUCUUUACUAGAGGUAUCCGA; SRSF11^#2^ Sense: AGAUCAAGAUCACGUUCUAGG, Anti‐Sense: UAGAACGUGAUCUUGAUCUUG; SRSF11^#3^ Sense: GCAAGAAGAAGAAGAGUAAAG, Anti‐Sense: UUACUCUUCUUCUUCUUGCUU. The sequence of HSPA12A‐exon 2‐specific siRNAs are as follows: Sense: UCAUCAUGGAGGAAGAAGGCG, Anti‐Sense: CCUUCUUCCUCCAUGAUGAAA. The Flag‐mCherry‐SRSF11 and MYC‐PAK5 were constructed with the pcDNA3.1(+) vector from Shanghai Hewu Biotechnology Co. Ltd. The lentiviruses packaged with SRSF11 (NM_004768.5) plasmids and empty vectors were produced by GenePharma. To generate stable strains, cells that had been transfected with lentiviruses for 72 h with the help of polybrene (10 µg/ml) were administrated with puromycin (5 µg/ml).

### 2.2 Tissue samples

Forty‐six pairs of CRC and normal adjacent tissues (Cohort 1) were acquired from CRC patients experiencing surgery at the Affiliated Hospital of Xuzhou Medical University with written informed consent. The resected tissues were rapidly frozen at −80°C for subsequent experiments. Seventy‐seven pairs of CRC tissue microarray chips were customized from TuFeiBio Co. Ltd.

### 2.3 Immunohistochemistry

After deparaffinization, rehydration and antigenic retrieval, sections were sent to implement antigen repair via the thermal repair method. Then the sections were sent for further H&E staining or antibody incubation according to standard procedure. Halo Version 3.0.311.314 software was applied for analysis following scanning with a PANNORAMIC panoramic slice scanner. The following formula was used to determine the score: Σ (positive rate × intensity).

### 2.4 Site‐directed mutagenesis of the phosphorylation site and plasmid construction

The plasmids were constructed from IBSBIO. The wild‐type or mutant Flag‐SRSF11 plasmids were recombinant plasmids constructed by inserting the human SRSF11 gene (NM_004768.5) or the respective mutants into the pcDNA3.1(+) empty vector plasmid. Therein, S249/277/287/295/348A means the serine (S) of 249/277/287/295/348 site was mutated into alanine (A) by site‐directed mutagenesis.

### 2.5 qRT‐PCR, RT‐PCR and Western blotting

Total RNA was isolated by Trizol Reagent (Vazyme, catalog: R401‐01), reverse transcribed into cDNA by First Strand cDNA Synthesis Kit (Servicebio, catalog: G3330‐50) and utilized as the template for amplification with SYBR Green qPCR Master Mix (Servicebio, catalog: G3322‐05). The primers of qRT‐PCR were as follows: HSPA12A‐Intron 1, Forward: ATGTGCCTTGGGGAACCAG, Reverse: CAGGAAGCGTTGTGTGCAC; HSPA12A‐Intron 1‐Exon 2, Forward: GTGCACACAACGCTTCCTG, Reverse: CCTTGGGCAAGGTGCTTAG; HSPA12A‐Exon 2‐Intron 2, Forward: ATGAAAACATCTGAGACTCAGGG, Reverse: CGTACTCGTGCTGGTTGAC; SRSF11, Forward: GACTAATGTCTCCCCGAGCG, Reverse: AGACTGGCAAAGGCGAATCA; N‐cadherin, Forward: GTGCATGAAGGACAGCCTCT, Reverse: CCGTGGCTGTGTTTGAAAGG. Amplification of DNA was performed by Fast sTaq PCR Master Mix (Servicebio, catalog: G3304‐05) following the guidelines provided by the manufacturers. The primers of RT‐PCR were as follows: HSPA12A, Forward: TAGAAGGTGGAGTTGAGCAG, Reverse: TGAAGCGGCTATTTCCCAC. Relative quantitation of RNA was normalized to GAPDH levels; GAPDH, Forward: GTCAGTGGTGGACCTGACCT, Reverse: TGCTGTAGCCAAATTCGTTG.

RIPA lysis buffer (KeyGEN BioTECH, catalog: KGP702) was used to extract all the protein, and Enhanced BCA Protein Assay Kit (KeyGEN BioTECH, catalog: KGP903) was used for quantification. Nuclear extraction kit (KeyGEN BioTECH, catalog: KGP826) was applied for the isolation and extraction of cytoplasmic and nuclear proteins. Equivalent protein was separated by 10% SDS‐PAGE electrophoresis, followed by transmembrane using the nitrocellulose filter membranes (Pall, catalog: 66485). Then the membranes were blocked by 5% non‐fat dry milk or BSA for 2 h at room temperature and followed by the corresponding antibodies incubation. GAPDH, β‐actin or PCNA were used as internal controls. TanonTM High‐sig ECL Western Blot Substrate (Tanon) was used to reveal protein bands, followed by analytical processing of Tanon's Image Analysis Software. Then, the gray value of all bands was calculated by the software ImageJ.

### 2.6 Mn^2+^‐Phos‐tag SDS‐PAGE

Dephosphorylation of phosphorylated proteins was acquired from a 50 mM Tris‐HCl buffer (pH 9.0) containing 1.0 mM MgCl_2_, 30 µg of protein and 3.3 units of AP, followed by incubation at 37°C for 12 h and then mixed with 100 µl of 3× SDS‐PAGE loading buffer. For Mn^2+^‐Phos‐tag SDS‐PAGE, acrylamide‐pendant Phos‐tag ligand (50 µM) and two equivalents of MnCl_2_ were added to the separating gel before polymerization. An acrylamide stock solution was prepared as a mixture of a 29:1 ratio of acrylamide to *N,N*′‐methylenebisacrylamide. The electrophoresis running buffer (pH 8.4) was 25 mM Tris and 192 mM glycine containing 0.1% (w/v) SDS as in normal SDS‐PAGE. Before a typical SDS‐PAGE transmembrane, the gel undergoing electrophoresis was washed modestly in 1 mmol/L EDTA for 10 min, followed by gentle washing in a general transfer buffer without EDTA for 10 min. Then, the gel was transmembrane using the PVDF membranes followed by a block for 2 h at room temperature and incubated with specific primary antibodies and the following secondary antibodies as in normal Western blotting (WB) analysis.

### 2.7 In vitro phosphorylation assay

Phosphorylation assay was performed with a Homogeneous Time‐Resolved Fluorescence (HTRF) KinEASE‐STK (serine/threonine kinase) S2 kit (Cisbio, France) as per the guidelines provided by the manufacturer. The KinEASE‐STK S2 kit is suitable for measuring the activities of serine/threonine kinases, including PAK5. Briefly, the reagents needed for the assay were prepared following the instructions. The 1× kinase buffer was prepared by distilled water dilution of 5× buffer supplemented with DTT (1 mM; Sigma‐Aldrich) and MgCl_2_ (5 mM, Sigma). The STK substrate 2‐biotin and the synthetic peptides were treated in a 50 µM solution with distilled water. Streptavidin‐XL665 was diluted to 500 nM. Recombinant human PAK5 protein (Abcam, catalog: ab60754) was stored at −80°C. The detection buffer was resuspended in distilled water and employed to formulate the working solution of Streptavidin‐XL665 and STK‐antibody‐cryptate. Adenosine triphosphate (Sigma) was diluted to 5 mM with 1× kinase buffer.

The assay was performed with an HTRF 96‐well low‐volume plate (Cisbio, catalog: 66PL96001). Next, 4 µl of 1× kinase buffer, 2 µl of STK substrate 2‐biotin or synthetic peptide solution, 2 µl of recombinant PAK5 protein (0.1 mg/ml) and 2 µl of ATP were added in turn to 96‐well plates. After sealing the plate and incubating for 30 min at 37°C, 5 µl of STK antibody‐cryptate and Streptavidin‐XL665 were added, respectively. After 1 h of incubation, the acceptor and donor emission signals of each individual well were detected by a VICTOR Nivo multimode microplate reader (PerkinElmer, Waltham, MA, USA). The relative phosphorylation level was converted according to the ratio of (signal 665 nm/signal 620 nm) ×10^4^.

### 2.8 Transwell and xenograft assays

For the migration and invasion assays, Transwell inserts (Corning Incorporated) without or with Matrigel (BD Biosciences) coating were used. A 24‐well plate containing inserts of 8 µm size was put in place, and each well was prefilled with 480 µl of culture medium together with 120 µl FBS. Then, each insert was filled uniformly with 100 µl of 5 × 10^4^ cell suspension. Paraformaldehyde (Servicebio, catalog: G1101) was applied to fix cells for 20 min, followed by a 24‐h culturation, and then the cells were stained using 0.1% crystal violet (Servicebio, catalog: G1014) for 15 min. Cells that did not reach the chamber's upper layer were wiped off after being thoroughly cleaned with cotton swabs and washed with PBS. Then, we used a Nikon digital camera to take pictures with a magnification of ×200 followed by the software, ImageJ (Version 1.53, National Institutes of Health, Bethesda, MD, USA) to compute the number of cells penetrating through the pores.

Five‐week‐old, male nude mice (BALB/c) that were acquired from Nanjing GemPharmatech Co., Ltd were used to construct the xenograft tumour formation assay. The mice were intravenously inoculated via the tail veins with the stable strains (NC or SRSF11^OE^) (5 × 10^6^ cells suspended in 100 µl PBS). In accordance with the Institutional Animal Care and Use Committee's rules, all animals were raised in conditions free of pathogens. Six weeks later, mice were euthanased, and the isolated lung tissues were resected and used for subsequent experiments.

### 2.9 Immunofluorescence

The cells were fixed using paraformaldehyde for 20 min at room temperature, followed by a 30‐min treatment of 0.5% Triton X‐100 (Sigma‐Aldrich). Goat serum (ZSGB‐BIO) was used for blocking for 30 min. The cells were incubated with the anti‐Flag primary antibody (1:200, Proteintech, catalog: 20543‐1‐AP) or anti‐N‐cadherin primary antibody (1:200, Abclonal, catalog: A0432) overnight at 4°C, and subsequently with the respective secondary antibody conjugated with CoraLite488 (1:100, Proteintech, catalog: SA00013‐2) after being washed thrice in PBST (PBS with 1‰ Triton X‐100). Phalloidin (1:100, Proteintech, catalog: PF00003) staining was used to observe the actin cytoskeleton. The DNA dye DAPI (Biosharp, catalog: BL105A) was applied for nucleus staining. Fluorescent scanning analysis was employed through the STELLARIS 5 confocal fluorescence microscope (Leica, China) under established methods.

### 2.10 Coimmunoprecipitation

The coimmunoprecipitation (Co‐IP) method was carried out via the Protein A+G Agarose (Beyotime Biotechnology, catalog: P2019). Collected cells were resuspended in IP lysis buffer (Beyotime Biotechnology, catalog: P0013) for a 30‐min lysis, followed by a 15‐min centrifugation at 12 000 × *g* and 4°C. The supernatants were incubated with the indicated antibody (2 µg per 500 µg of total protein in 1 ml of cell lysate) overnight at 4°C, followed by 2 h of incubation with 50 µl Protein A+G Agarose beads at 4°C. The suspension was collected by centrifugation followed by extensive washing with IP lysis buffer. After then, the sediment was dissolved in 30 µl of 1× SDS loading buffer (Generay Biotech, catalog: GR0208). The antibodies used are listed as follows: SRSF11 (Abcam, catalog: ab254733), PAK5 (Bioworld, catalog: BS9193), MYC (Proteintech, catalog: 16286‐1‐AP) and Flag (Proteintech, catalog: 20543‐1‐AP).

### 2.11 in vivo crosslinking followed by immunoprecipitation

The in vivo crosslinking and immunoprecipitation (CLIP) assay was performed according to the previous description with fine‐tuned adjustments.^27^ Shortly, cells cultured in a 10‐cm dish were harvested and cross‐linked under ultraviolet. Then IP procedure was conducted by Flag‐tag polyclonal antibody (catalog number: 20543‐1‐AP) or rabbit IgG polyclonal antibody (catalog number: 30000‐0‐AP) from Proteintech Group. Cell extracts were incubated with indicated antibodies overnight at 4°C followed by coincubation with 50 µl magnetic beads (MCE, catalog number: HY‐K0205) per pipe for 1 h. The next step was to clean the beads three times by washing buffer with 1% cocktail and 1 U/µl RNase inhibitor (Abclonal, catalog number: RK21401) and suspending in 120 µl elution buffer with 1% cocktail and 1 U/µl RNase inhibitor at 30°C for 15 min. Then another Eppendorf tube was used to collect the supernatant, and 5 µl 4.8 M NaCl with 1 U/µl RNase inhibitor was added followed by shaking overnight at 65°C. Proteinase K was used to digest the protein therein at 60°C for 1 h. Following RNA extraction and reverse transcription with random primers, PCR assays were performed using specially designed primers to amplify the skipped cassette as well as the flanking exons.

### 2.12 RNA‐seq and data analysis

Following the manufacturer's guidelines, 150 bp paired‐end RNA‐seq using an Illumina NovaSeq platform was performed on total RNA isolated from LoVo cells transfected with negative control (si‐NC) and si‐SRSF11, and rMATS (3.2.5) software was used to analyze the differential AS events. The rMATS software can classify the AS events into five categories (ES, intron retention, alternative 5′ splice site, alternative 3′ splice site and mutually exclusive exon), and can conduct differential AS analysis on samples with biological duplication. Each AS event corresponds to two isoforms, which are exon inclusion (EI) isoform and ES isoform. The expression level of the two isoforms is counted and divided by their effective length to receive the accommodated expression level. The ratio of the total expression amount of EI isoform in the two isoforms is calculated, namely IncLevel. Finally, the significance of the differences was analyzed. Under the accession number of GSE199094, the raw sequence data have been uploaded to the Gene Expression Omnibus.

### 2.13 Motif analysis of SRSF11‐mediated alternative exons

All sequences of skipping exon events regulated by SRSF11 according to the RNA‐seq data described above were sorted out by FASTA format. The sequence alignment was then performed using Jalview Version 2.11.2.0 software. The aligned base was visualized according to the sequence consensus, in which the topmost represents a larger proportion and the bottommost represents a smaller proportion.

### 2.14 TCGA RNA‐seq data analysis

RNA‐seq data (RPKM) from 434 COAD and READ and normal samples were obtained from the TCGA database. The correlation scatter plot of SRSF11 expression levels between tumour and normal tissues was generated by GraphPad Prism 9. The correlation scatter plot between SRSF11 and N‐cadherin was downloaded from the TCGA database.

### 2.15 Mini‐gene reporter assay

Mini‐genes were established according to the previous description.^28^ In short, the HSPA12A‐WT mini‐gene was generated by cloning a sequence containing exons 1–3 together with 300 bp at both ends of introns 1–2 into the pcDNA3.1(+) vector. Using the HSPA12A‐WT mini‐gene plasmid as a base, site‐mutation versions or deletions were created, in which the deletion plasmid (HSPA12A‐ΔEx2) was deleted with exon 2 sequences, and the natural GACTCA sequence of HSPA12A‐WT in exon 2 was randomly site‐mutated (HSPA12A‐Mut) by online tools.

### 2.16 Statistical analysis

SPSS 26.0 was applied for the statistical analysis, and GraphPad Prism 9 was utilized to visualize all results. Data were presented as mean value ± SD after three times repeating each experiment. Parametric comparisons were conducted with Student *t* test, Student *t*′ test, or Student–Neuman–Keuls (SNK) tests. Non‐parametric Mann–Whitney *U* tests or Kruskal–Wallis tests were followed with non‐parametric Wilcoxon two‐group tests. The associations between SRSF11 and CRC patients’ clinical‐pathological characteristics were performed by two‐sided Fisher's exact tests. In addition, survival curves were evaluated by Kaplan–Meier methods, with comparisons operated using the log‐rank test. For correlation, Pearson correlation test was used. *p* < .05 was defined as statistically significant.

## 3 RESULTS

### 3.1 SRSF11 expression is elevated in CRC and is associated with clinical‐pathological characteristics and prognosis in CRC patients

**FIGURE 1:**
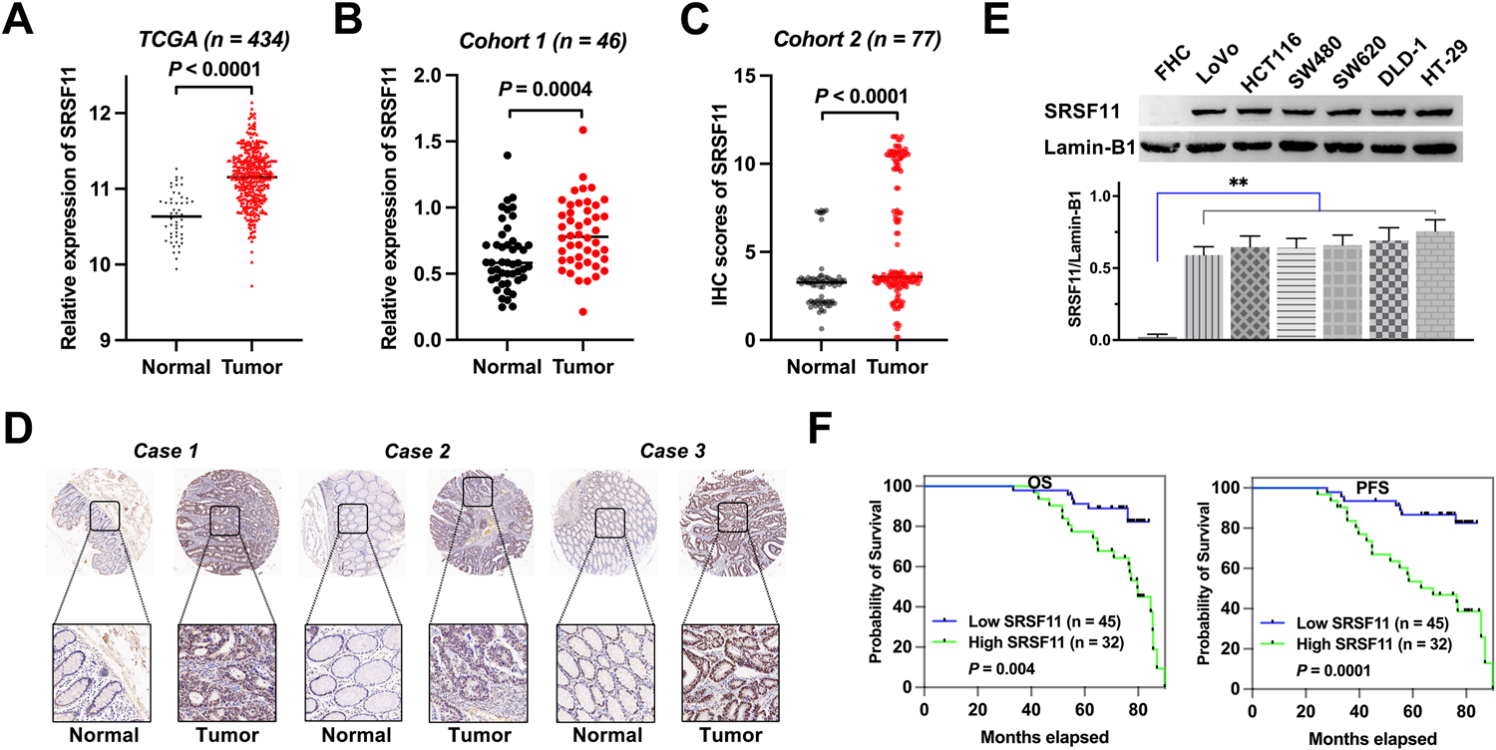
SRSF11 is upregulated in CRC and clinically relevant to prognostic significance. (A and B) Relative expression of SRSF11 in paired colorectal cancer and matched normal tissue samples from (A) TCGA‐seq dataset (Mann–Whitney test, *p* < .0001). (B) Forty‐six pairs of colorectal cancer and matched normal tissue samples from the Affiliated Hospital of Xuzhou Medical University (paired *t*‐test, *p* = .0004). (C) IHC scores of SRSF11 in CRC tissue chip (*N* = 77; paired *t*‐test, *p* < .0001). (D) The representative IHC images of SRSF11 in three pairs of CRC tissues obtained from tissue chip. (E) WB analysis of the protein level of SRSF11 in normal colonic epithelial FHC cells and six malignant CRC cell lines (Kruskal–Wallis test, **p < .01). (F) Kaplan–Meier plots of CRC patients with high SRSF11 expression against low SRSF11 expression (log‐rank; OS, *p* = .004; PFS, *p* = .0001). OS, overall survival; PFS, progression‐free‐survival; CRC, colorectal cancer; TCGA, The Cancer Genome Atlas

To evaluate SRSF11's expression levels in CRC, we analyzed a public database from The Cancer Genome Atlas (TCGA) and found that the gene expression of SRSF11 was significantly higher in CRC compared with normal tissues, with a statistically significant difference (*p* < .0001) (Figure 1A). Based on the findings from the TCGA database, we investigated the differences in SRSF11 expression between the tumour and normal adjacent tissues from clinical CRC patients undergoing surgical resection. Forty‐six pairs of CRC tumours and normal adjacent tissues (Cohort 1) were collected from the Affiliated Hospital of Xuzhou Medical University followed by WB analysis. The findings indicated that SRSF11 expression levels are considerably higher in CRC tissues than those in normal tissues (*p* = .0004) (Figure 1B and Figure S1). Additionally, we conducted an immunohistochemistry (IHC) experiment with a tissue microarray chip containing 77 pairs of CRC tumour and normal tissue points (Cohort 2). The IHC scores were significantly higher in tumour tissues than in normal tissues (*p* < .0001) (Figure 1C), and three typical paired tissues are shown in Figure 1D. We further assessed the protein level of SRSF11 in CRC cell lines by WB analysis, and the results showed that the six CRC cell lines had higher SRSF11 expression levels than normal intestinal epithelial FHC cells (Figure 1E).

Furthermore, the clinical‐pathological characteristics of the 77 pairs of CRC samples are displayed in Table 1. Two‐sided Fisher's exact test revealed that high SRSF11 expression was positively correlated with TNM stage (*p* < .0001), pN status (*p* = .011), pM status (*p* < .0001) and recurrence (*p* = .008). Additionally, Kaplan–Meier analysis revealed that patients with higher levels of SRSF11 possessed inferior OS and progression‐free survival (PFS) than those with lower levels (*p* = .004; *p* = .0001) (Figure 1F). Together, these findings show that SRSF11 is increased in CRC and that this overexpression is associated with clinical‐pathological characteristics and prognosis of CRC patients.

**TABLE 1:** The clinical characteristics of 77 pairs of biopsies from colorectal cancer patients

**Variables**	**SRSF11 staining**			
	**Low** ^a^	**High** ^b^	**Total**	***p*** ^c^
**Age**				
≤64 years	27	13	40	.148
>64 years	18	19	37	
**Gender**				
Male	19	16	35	.658
Female	26	16	42	
**pT status**				
pT_1_–pT_3_	24	17	41	.986
pT_4_	21	15	36	
**pN status**				
pN_0_	35	15	50	**.011**
pN_1_–pN_3_	10	17	27	
**pM status**				
pM_0_	40	11	51	**.000**
pM_1_	5	21	26	
**TNM stage**				
I–III	40	11	51	**.000**
IV	5	21	26	
**Tumour recurrence**				
No	39	18	57	**.008**
Yes	6	14	20	

^a^ IHC score 0–8.

^b^ IHC score >8.

^c^ Two‐sided Fisher's exact tests.

### 3.2 SRSF11 exhibits a pro‐metastatic effect in vitro and in vivo in CRC

**FIGURE 2:**
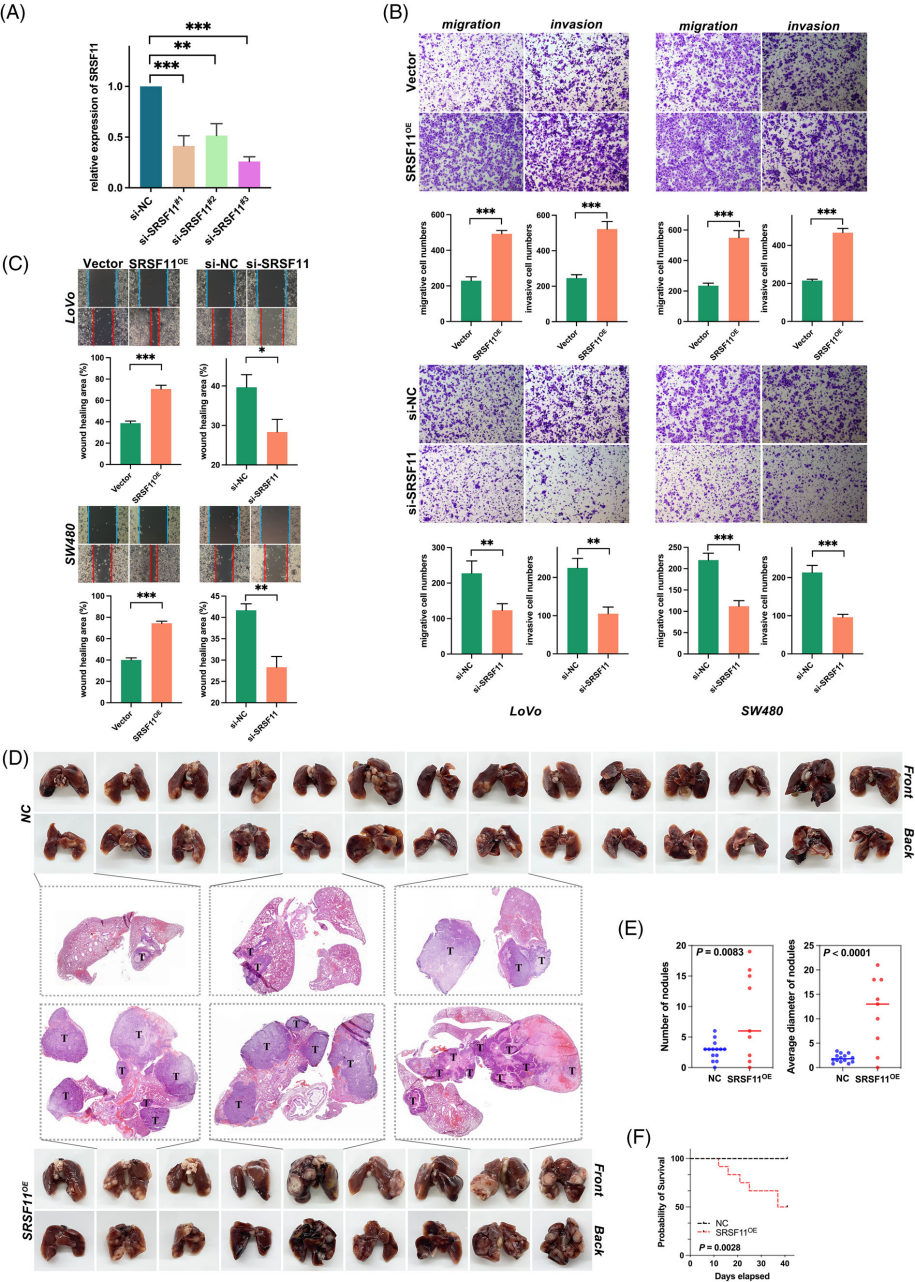
SRSF11 promotes CRC migration and invasion in vitro and metastasis in vivo. (A) qPCR analysis of SRSF11 mRNA in negative control (si‐NC) and three sets of small interfering RNA for SRSF11 (si‐SRSF11^#1‐3^) (Kruskal–Wallis test, **p < .01, ***p < .001). (B) Transwell assay for investigating migration and invasion capacities in SRSF11 overexpression (SRSF11^OE^), si‐SRSF11 as well as the negative control (Vector, si‐NC), respectively (*N* = 3; Student *t*′ test; **p < .01, ***p < .001). (C) Representative images of the cell wound healing after transfection with SRSF11^OE^ or si‐SRSF11, as well as the corresponding negative control (*N* = 3; Student *t*′ test; *p < .05, **p < .01, ***p < .001). (D) Images of the lungs of mice 6 weeks following injections of NC and SRSF11^OE^ group cells. The H&E staining in pulmonary metastatic foci is shown in the middle, with arrows indicating the metastatic foci. (E) Quantification of the numbers of nodules or their average diameter in both groups (*N* = 14 in NC group, *N* = 9 in SRSF11^OE^ group; Student *t* test). (F) Kaplan–Meier plots of mice in NC and SRSF11^OE^ groups (*N* = 15, for each, log‐rank)

**FIGURE 3:**
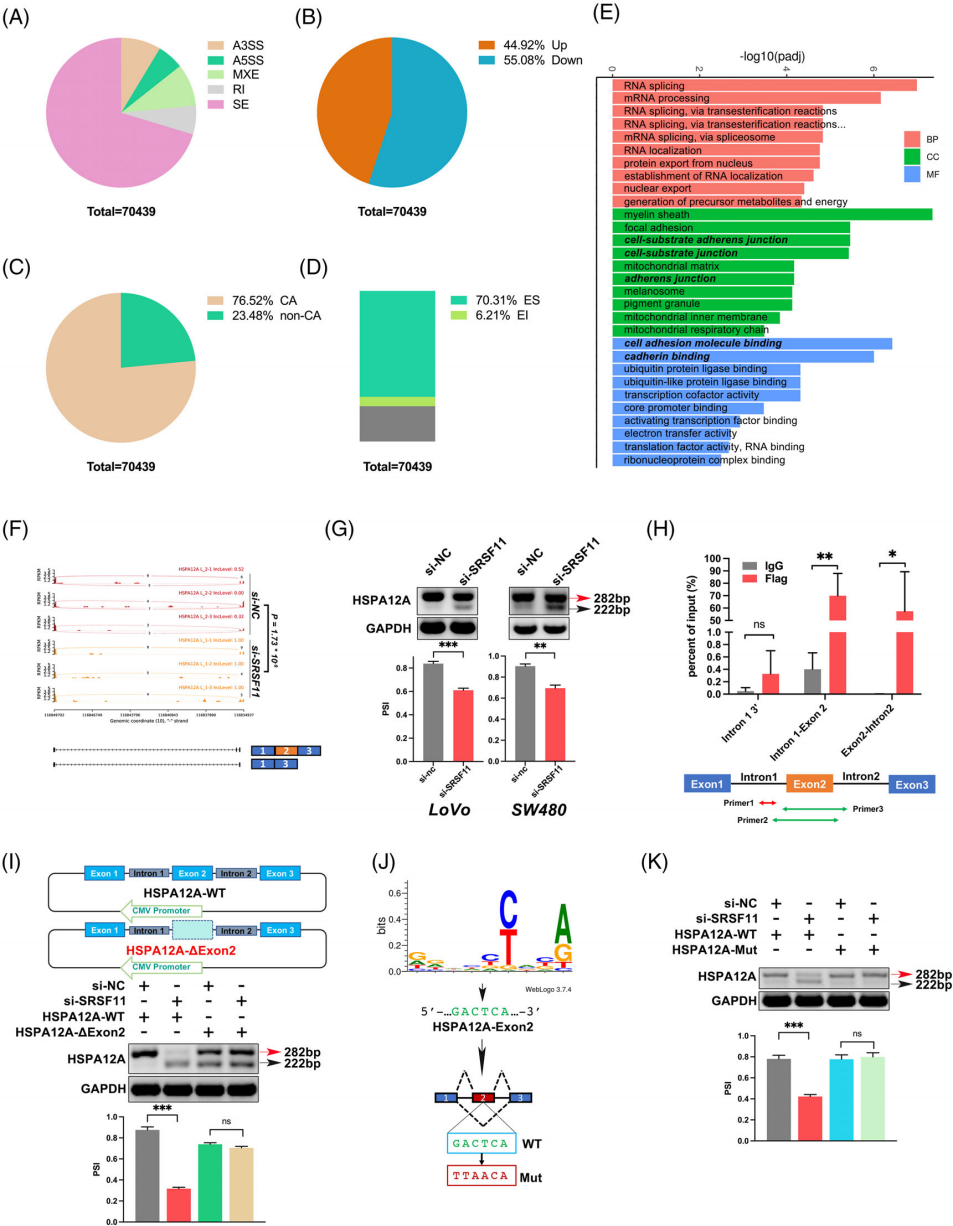
AS and transcriptome profiles regulated by SRSF11 in LoVo cells. (A–D) The proportion regulated by SRSF11 classified by AS types (A), PSI value (B), cassette exons AS (C and D). (E) Gene ontology of the differentially expressed genes between si‐NC and si‐SRSF11 LoVo cells. |LogFC| > 1, FDR < 0.05. (F) SRSF11 regulated HSPA12A pre‐RNA splicing and promoted the inclusion of HSPA12A exon 2. (G) SRSF11 triggered SE events of the HSPA12A pre‐RNA. RT‐PCR results and quantification of their RNA products were measured as inclusion/(inclusion+exclusion) (PSI) below. Note that alternative exon 2 for SRSF11‐mediated inclusion is marked in red while SRSF11‐mediated exclusion is marked in black arrow (*N* = 3, Student *t*′ test; *p < .05, **p < .01). (H) CLIP‐qPCR analysis of SRSF11 association with HSPA12A pre‐RNA at the indicated locations. IgG, control immunoglobulin (*N* = 3, Student *t*′ test; ns, no significance, *p < .05, **p < .01). (I) The top shows the schematic diagram of the HSPA12A mini‐gene constructs (HSPA12A‐WT and HSPA12A‐ΔExon 2). The bottom exhibits the in vivo splicing analysis of HSPA12A mini‐gene and indicated deletion mutants in LoVo cells. Quantification analysis is shown below (*N* = 3, SNK test; ns, no significance, ***p < .001). (J) Deduced SRSF11 binding consensus based on the SE events from our RNA‐seq results. The green font represents the matched sequences within exon 2 of HSPA12A, and the red font indicates the random mutation. (K) in vivo splicing analysis of HSPA12A mini‐gene and indicated deletion mutants in LoVo cells. Quantification analysis is shown below (*N* = 3, SNK test; ns, no significance, ***p < .001).

To fully examine the biological role of SRSF11 in CRC in vitro, we knocked down SRSF11 in the LoVo cell line, and qRT‐PCR analysis determined the knockdown efficiency (Figure 2A). Then, we performed RNA‐seq using si‐SRSF11 and the control si‐NC LoVo cell lines followed by gene ontology (GO) analysis and found that SRSF11 was engaged in cell–substrate adherens junction and cadherin binding, indicating its role in epithelial–mesenchymal transition (EMT) progression (Figure 3E). Transwell migration/invasion and wound healing experiments demonstrated that overexpression of SRSF11 might enhance the capacity for migratory and invasive properties in both LoVo and SW480 cell lines, and its knockdown significantly restrained the migration and invasion capacities (Figure 2B,C).

To explore the role of SRSF11 in CRC metastasis in vivo, we injected nude mice with SRSF11 stable‐overexpression (SRSF11^OE^) and the corresponding control (NC) cells through tail vein injection and assessed lung metastatic potential 6 weeks later. H&E staining revealed that the SRSF11^OE^ group had more lung metastases than the NC group (Figure 2D,E). More notably, when compared with NC, SRSF11 overexpression dramatically reduced mouse survival (Figure 2F). These findings suggest that SRSF11 enhances the potential of CRC metastasis.

### 3.3 Identification of AS events regulated by SRSF11

The role of SRSF11‐related AS events in carcinogenesis and progression remains unclear. We applied next‐generation RNA‐seq to analyze three pairs of independent RNA samples from control and SRSF11‐knockdown LoVo cells and identified a total of 70 439 SRSF11‐regulated AS events (Figure 3A). Under the knockdown of SRSF11, the index of percent spliced in (PSI) was upregulated in 31 638 (44.92%) AS events and downregulated in 38 801 (55.08%) AS events (Figure 3B). More than 76% (53 900/70 439) of these AS events are related to cassette exons (CA) (Figure 3C). CA AS contains EI and ES. In our investigation, SRSF11 knockdown caused 49 523 (70.3%) ES and 4377 (6.2%) EI alterations (Figure 3D), indicating that SRSF11 plays a dominating role as a trigger of ES events.

ClueGO analysis of SRSF11‐regulated genes indicated probable biological roles for SRSF11, and their molecular function was enriched for adherens junction‐associated pathways (Figure 3E). Furthermore, the differential AS analysis revealed that SRSF11 knockdown dramatically elevated the expression of HSPA12A (ENST00000453491.3) exon 2‐inclusive splice variants (Figure 3F). And the results were validated by PSI values by applying RT‐PCR with designed primers targeting exons 1–3 of HSPA12A in both LoVo and SW480 cell lines (Figure 3G). To discuss the binding motifs of SRSF11 in HSPA12A pre‐RNA, we conducted RNA immunoprecipitation (RIP) assays and discovered that SRSF11 roughly bound with HSPA12A within exon 2‐centred motifs (Figure 3H). To explore the potential significance of exon 2 in the SRSF11‐regulated AS, we constructed mini‐genes of HSPA12A genomic DNA that contain exons 1–3 with 300 bp flanking introns (HSPA12A‐WT) and mini‐genes of HSPA12A genomic DNA with deletion of exon 2 sequence compared with HSPA12A‐WT (HSPA12A‐ΔEx2). Consistent with the data from the endogenous genes, the knockdown of SRSF11 decreased the PSI of the pre‐RNA transcribed from the HSPA12A‐WT mini‐genes to the HSPA12A transcripts, whereas the PSI remained unchanged with HSPA12A‐ΔEx2 mini‐genes, indicating that the exon 2 is required for SRSF11‐regulated splicing (Figure 3I). To define the particular binding motifs of SRSF11 in HSPA12A exon 2, we performed motif analysis of all CA (ES and EI) events using Jalview software and found that SRSF11 appears to prefer regular sequences. Surprisingly, we discovered that the GACTCA 6‐mer motif of HSPA12A exon 2 coincided with the preference sequences (Figure 3J). To determine whether it is required for SRSF11‐regulated splicing, we randomly altered the 6‐mer motif via an online random mutator tool (http://www.detaibio.com/sms2/mutate_dna.html)^29^ followed by a mini‐gene assay in which the GACTCA sequence was mutated into TTAACA (HSPA12A‐Mut). Surprisingly, the mini‐gene reporter assay validated that the GACTCA motif was vital for SRSF11 splicing on HSPA12A pre‐RNA (Figure 3K). Taken together, these findings show that SRSF11 modulates HSPA12A exon 2 ES via binding to its GACTCA motif.

### 3.4 Silencing HSPA12A‐Ex2+ antagonized SRSF11‐induced CRC migration and invasion phenotypes in vitro by suppressing EMT progression

**FIGURE 4:**
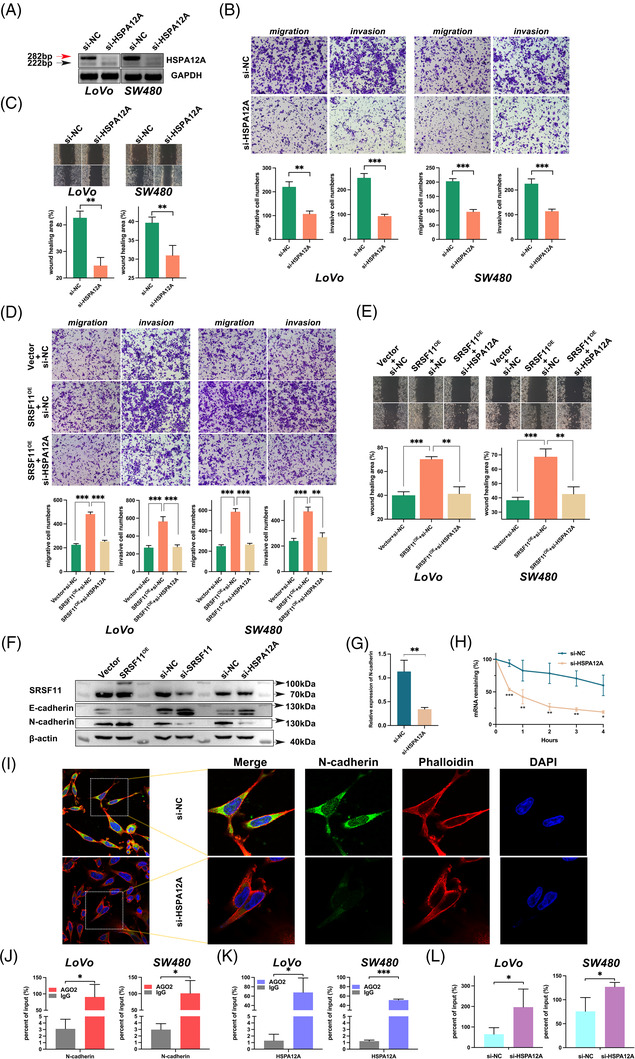
SRSF11 promotes cell migration and invasion via HSPA12A‐Ex2+ stabilizing N‐cadherin mRNA in vivo. (A) Confirmation of the RNA levels of two HSPA12A transcripts after knockdown of HSPA12A treatments in LoVo and SW480 cell lines. (B) The cell migration and invasion of LoVo and SW480 cells after transfection with si‐HSPA12A and the negative control were detected by Transwell assays (*N* = 3; Student *t*′ test; **p < .01, ***p < .001). (C) Representative images of the cell wound healing after transfection with si‐HSPA12A and respective negative control (*N* = 3; Student *t*′ test; **p < .01). (D) Transwell assay for investigating the abilities of migration and invasion in LoVo and SW480 cells after co‐transfection with indicated plasmid and siRNA (*N* = 3; Student *t*′ test; **p < .01, ***p < .001). (E) Representative images of the cell wound healing of shift after co‐transfection with indicated plasmid and siRNA. The statistical diagrams were displayed below (*N* = 3; Student *t*′ test; **p < .01, ***p < .001). (F) WB analyses of EMT markers, E‐cadherin and N‐cadherin in LoVo cells after treatment with SRSF11 overexpression or knockdown of SRSF11 or HSPA12A. (G) qPCR detection of N‐cadherin mRNA after treatment with si‐HSPA12A (*N* = 3; Student *t*′ test; **p < .01). (H) RNA stability detection between si‐HSPA12A and si‐NC group by ActD treatment for 4 h (*N* = 3; SNK test, *p < .05, **p < .01, ***p < .001). (I) Representative immunofluorescence images by confocal microscope indicate N‐cadherin (green), Phalloidin (red) and DAPI (blue) localization in LoVo cells. Phalloidin staining was used to observe the actin cytoskeleton. (J) RIP experiments of N‐cadherin RNA by AGO2 antibody in LoVo and SW480 cell lines (*N* = 3; Student *t*′ test; *p < .05). (K) RIP experiments of HSPA12A RNA by AGO2 antibody in LoVo and SW480 cell lines (*N* = 3; Student *t*′ test; *p < .05, ***p < .001). (L) RIP experiments of N‐cadherin RNA by AGO2 antibody after treatment with si‐NC or si‐HSPA12A in LoVo and SW480 cell lines (*N* = 3; Student *t*′ test; *p < .05)

Given that SRSF11 is implicated in CRC metastasis and promotes HSPA12A exon 2 inclusion, we hypothesized that HSPA12A exon 2‐inclusive transcript (HSPA12A‐Ex2+) could contribute to CRC metastasis. To verify this hypothesis, we designed a set of siRNAs selectively targeting the exon 2 of HSPA12A (si‐HSPA12A) and RT‐PCR validated the efficiency of interfering HSPA12A‐Ex2+ transcript in both LoVo and SW480 cell lines (Figure 4A). Then, we performed Transwell assays and found that si‐HSPA12A prominently inhibited the abilities of cell migration and invasion as well as wound healing in both LoVo and SW480 cells, as expected (Figure 4B,C). Given that SRSF11 promotes the production of HSPA12A‐Ex2+ transcript and that knockdown of HSPA12A‐Ex2+ significantly inhibits CRC cell migration and invasion, we hypothesize that SRSF11 can influence CRC migration and invasion by increasing the splicing of HSPA12A‐Ex2+ transcripts. Rescue experiments were applied in Transwell as well as wound healing assays. As shown in Figure 4D,E, the enhancement of cell migration and invasion as well as wound healing abilities was dramatically counteracted after co‐transfection with si‐HSPA12A, indicating that HSPA12A‐Ex2+ mediated the enhancement of SRSF11‐induced CRC migration and invasion.

Because of the importance of EMT progression in tumour metastasis, we aimed to assess the impact of SRSF11 on E‐ and N‐cadherin expression levels, two important epithelial and mesenchymal gene markers of EMT progression. The results from WB analysis revealed that SRSF11 overexpression considerably decreased E‐cadherin protein levels while increasing N‐cadherin, whereas SRSF11 knockdown significantly reversed these changes (Figure 4F and Figure S2). We also captured that knockdown of HSPA12A‐Ex2+ enhanced E‐cadherin while decreasing N‐cadherin levels (Figure 4F and Figure S2). An online tool lncTar (http://www.cuilab.cn/lnctar) was applied to predict the possibility of HSPA12A‐Ex2+ interacting with E‐cadherin or N‐cadherin mRNA,^30^ and the results showed that the exon 2 of HSPA12A‐Ex2+ might bind with E‐cadherin or N‐cadherin mRNA with a low binding free energy (Figure S3). Further RNA stability tests with ActD revealed that HSPA12A‐Ex2+ considerably improved N‐cadherin mRNA stability (Figure 4G,H), but had no significant effect on E‐cadherin (data not shown). In addition, immunofluorescence (IF) analysis was conducted to confirm that the knockdown of HSPA12A significantly reduced the distribution of N‐cadherin in LoVo cell line (Figure 4I). These findings suggest that HSPA12A‐Ex2+ may aid in SRSF11‐induced CRC migration and invasion by boosting the stability of N‐cadherin mRNA via its exon 2 region.

To discuss the mechanism of HSPA12A‐exon 2 stabilizing N‐cadherin mRNA, we focused on the AGO2 protein, which catalyzes the RNA‐induced silencing complex (RISC) engine and guides gene silencing processes. RIP assays were conducted as shown in Figure 4J, and the results revealed that AGO2 interacts with N‐cadherin in both LoVo and SW480 cell lines. Intriguingly, the RIP assay showed that AGO2 also interacted with HSPA12A‐Ex2+, leading us to believe that HSPA12A‐Ex2+ may act as a transcriptionally competitive RNA for N‐cadherin (Figure 4K). To test this hypothesis, we downregulated HSPA12A‐Ex2+ and performed RIP assays, finding that the connection between N‐cadherin and AGO2 was greatly strengthened (Figure 4L). Further online prediction using catRAPID omics v2.0 (http://service.tartaglialab.com/page/catrapid_omics2_group)^31^ presented that AGO2 may interact with HSPA12A‐exon 2 and N‐cadherin on their 5′ area (Figure S4), which is consistent with the favourable fit of 5′‐C by AGO2.^32^ These findings imply that HSPA12A‐Ex2+ promotes SRSF11‐induced CRC migration and invasion by stabilizing N‐cadherin mRNA and inducing EMT progression.

### 3.5 Increased inclusion of HSPA12A‐exon 2 parallels enhanced SRSF11 expression in clinical CRC samples

**FIGURE 5:**
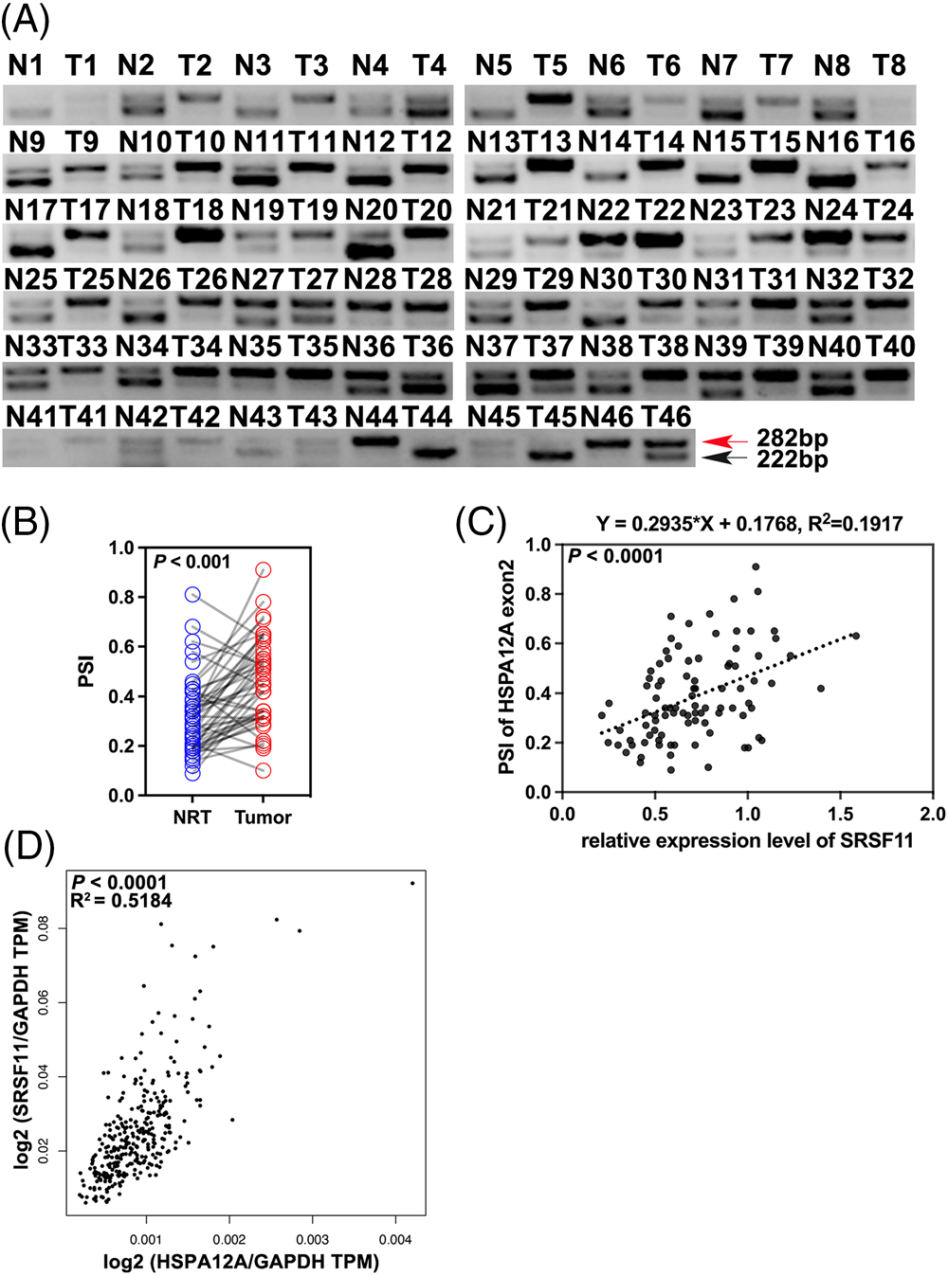
Increased inclusion of HSPA12A exon 2 parallels increased SRSF11 expression in clinical CRC samples. (A) RT‐PCR analysis of different HSPA12A transcripts in Cohort 1 samples. (B) The statistical diagram of A (*N* = 46; Student *t*′ test). (C) Correlation between PSI ratio and SRSF11 expression levels in Cohort 1 samples (Pearson correlation test). (D) Correlation between SRSF11 and HSPA12A expression levels in COAD and READ tissues from TCGA database (Pearson correlation test)

We next detected the PSI of HSPA12A‐exon 2 in 46 pairs of CRC and normal adjacent tissues. As shown in Figure 5A, the inclusion of exon 2 was significantly increased in tumour samples than in normal tissues (*p* < .001) (Figure 5B). Following that we used the relative expression of SRSF11 estimated in Cohort 1 to look for a possible link between HSPA12A‐exon 2 inclusion and SRSF11 protein levels. A positive association was observed between them with a *p*‐value <.0001 (Figure 5C). We corroborated this link further by analyzing the TCGA database on the GEPIA2 website (Figure 5D).^33^

### 3.6 Interaction between SRSF11 and PAK5

**FIGURE 6:**
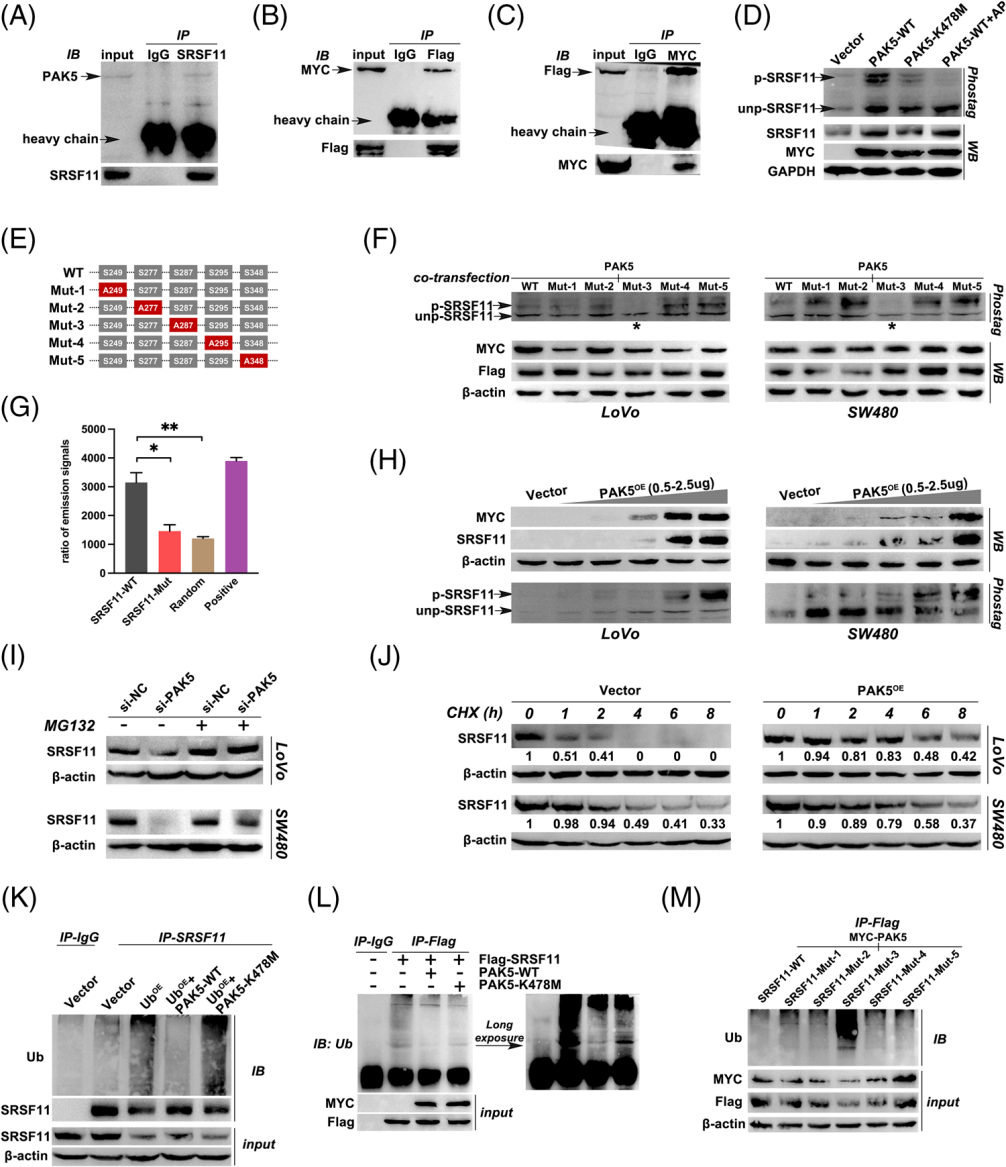
PAK5 phosphorylates SRSF11 at serine 287 site to protect it from ubiquitination degradation. (A) Immunoblotting analysis of endogenous PAK5 protein in LoVo cells using anti‐SRSF11. (B) Immunoblotting analysis of MYC‐tag PAK5 protein in LoVo cells co‐transfected with MYC‐PAK5 and Flag‐SRSF11 plasmids using anti‐Flag. (C) Immunoblotting analysis of Flag‐tag SRSF11 protein in LoVo cells co‐transfected with MYC‐PAK5 and Flag‐SRSF11 plasmids using anti‐MYC. (D) Phos‐tag SDS‐PAGE was performed in LoVo cells after transfection with Vector, PAK5‐WT, PAK5‐K478M or PAK5‐WT+AP as illustrated in Materials and Methods. p‐SRSF11 and unp‐SRSF11 represent the phosphorylated and unphosphorylated Slug, respectively. The total protein of SRSF11 was detected by WB analysis and shown below, with GAPDH serving as an internal control. (E) Schematic diagram of SRSF11 protein, wildtype (WT) or indicated mutants (serine replaced by alanine) fused with Flag. (F) Phos‐tag SDS‐PAGE was performed in LoVo and SW480 cells after co‐transfection with MYC‐PAK5 and WT or five mutants of Flag‐SRSF11 plasmids. The asterisks represent the significant reduction of p‐SRSF11. (G) in vitro phosphorylation assay was employed to explore the effects of PAK5 on different SRSF11 and control polypeptides. WT represents the wildtype SRSF11 polypeptides with 280–294 AA sequence; Mut represents the 284 serine replaced by alanine compared with WT; random represents that the 15 amino acids are randomly arranged in disorder; positive group represents the SRSF11 polypeptides replaced by PAK5 protein (*N* = 5, SNK test; *p < .05, **p < .01). (H) WB analysis of SRSF11 after gradient increasing overexpression of MYC‐PAK5 or Vector plasmid in LoVo and SW480 cell lines. The Phos‐tag SDS‐PAGE was performed to detect the p‐ and unp‐SRSF11 levels as shown below. (I) The effect of PAK5 on the stability of SRSF11. MG‐132 eliminates the effect of si‐PAK5 on the stability of SRSF11. (J) LoVo and SW480 cells were treated with CHX (50 nM) for 8 h after treatment with Vector or PAK5 plasmid. SRSF11 protein levels were determined at the indicated time points (the marked number below the strip represents the relative gray value to β‐actin in the same group). (K) Immunoblotting analysis of SRSF11 protein in LoVo cells after transfection with indicated plasmids using anti‐Ub. (L) Immunoblotting analysis of Flag‐tag protein in LoVo cells after transfection with indicated plasmids using anti‐Ub. (M) Immunoblotting analysis of Flag‐tag protein in LoVo cells after transfection with indicated plasmids using anti‐Ub

We wondered if SRSF11 could be phosphorylated by serine/threonine protein kinase PAK5 (Gene ID: 57144) because the RS domain is abundant in serine and arginine within serine/arginine‐rich family proteins. To clarify, we first performed Co‐IP analysis and confirmed that PAK5 interacted with SRSF11 at both endogenous (Figure 6A) and exogenous levels (Figure 6B,C). We then investigated whether phosphorylation regulation happened. Mn^2+^‐Phos‐tag SDS‐PAGE was carried out, and the shift in blotting indicated that wild‐type PAK5 overexpression (PAK5‐WT) could raise the expression level of phosphorylated SRSF11 (p‐SRSF11); however, the inactivated mutant PAK5 plasmid (PAK5‐K478M) was unable to increase p‐SRSF11 (Figure 6D). To avoid the false positive of gel shift, we additionally assessed p‐SRSF11 expression following treatment with AP, which can convert phosphorylated proteins to dephosphorylated status.^34^, ^35^ These findings indicated that PAK5 might phosphorylate SRSF11.

Given that PAK5 phosphorylates serine according to a set of regulations,^36^, ^37^ we examined the RS domain of SRSF11 and identified five possible phosphorylation sites, which were highlighted in red (Figure S5). To elucidate the location phosphorylated by PAK5 on SRSF11 amino acid (AA), we constructed a wildtype Flag‐SRSF11 plasmid (WT) and five mutated SRSF11 plasmids with five predicted serine mutated into alanine (Mut‐1/2/3/4/5), respectively (Figure 6E). After co‐transfection with PAK5 and these six plasmids, we detected the p‐SRSF11 levels by Mn^2+^‐Phos‐tag SDS‐PAGE. The blotting showed a distinct drop in the Mut‐3 group in both LoVo and SW480 cells, implying that the serine 287 (S287) was the trigger for PAK5 phosphorylation (Figure 6F). To strengthen this result, we applied an in vitro kinase assay using peptides synthesized as illustrated in Materials and Methods (Figure 6G). The results showed that when S287 was mutated, the PAK5 kinase activity shrunk considerably, confirming that PAK5 phosphorylated SRSF11 at S287.

Interestingly, PAK5 raised not only p‐SRSF11 but also unphosphorylated SRSF11 (unp‐SRSF11) (Figure 6D), indicating that PAK5 overexpression could increase the total SRSF11 protein levels. To clarify, we transfected PAK5 plasmids into LoVo and SW480 cells in a gradient‐increased dosage. As shown in Figure 6H, WB analysis revealed that PAK5 increased SRSF11 in a dose‐dependent manner. Further CHX and MG132 treatment confirmed that PAK5 may boost SRSF11 protein stability (Figure 6I,J); consequently, we focused on the latent regulation of PAK5 on the ubiquitination (Ub) degradation of SRSF11. An endogenous Ub‐IP experiment was performed in LoVo cells, and the results showed that PAK5 overexpression could drastically compromise the Ub level (Figure 6K). Exogenous Ub‐IP experiments showed that PAK5 rescued SRSF11 from Ub degradation (Figure 6L). Significantly, the preservation of SRSF11 protein stability may be related to PAK5 kinase activity, as PAK5‐K478M failed to attenuate Ub degradation of SRSF11 (Figure 6K,L). Notably, PAK5 phosphorylation on SRSF11 S287 was crucial for protein stability modulation, as S287 mutation (SRSF11‐mut3) resulted in excessive Ub levels, but the other groups showed normal Ub levels following co‐transfection with PAK5 plasmids (Figure 6M). Taken together, PAK5 could phosphorylate SRSF11 at S287, protecting SRSF11 against Ub degradation.

### 3.7 PAK5 facilitates the nuclear translocation and AS function of SRSF11 through its phosphorylation regulation

**FIGURE 7:**
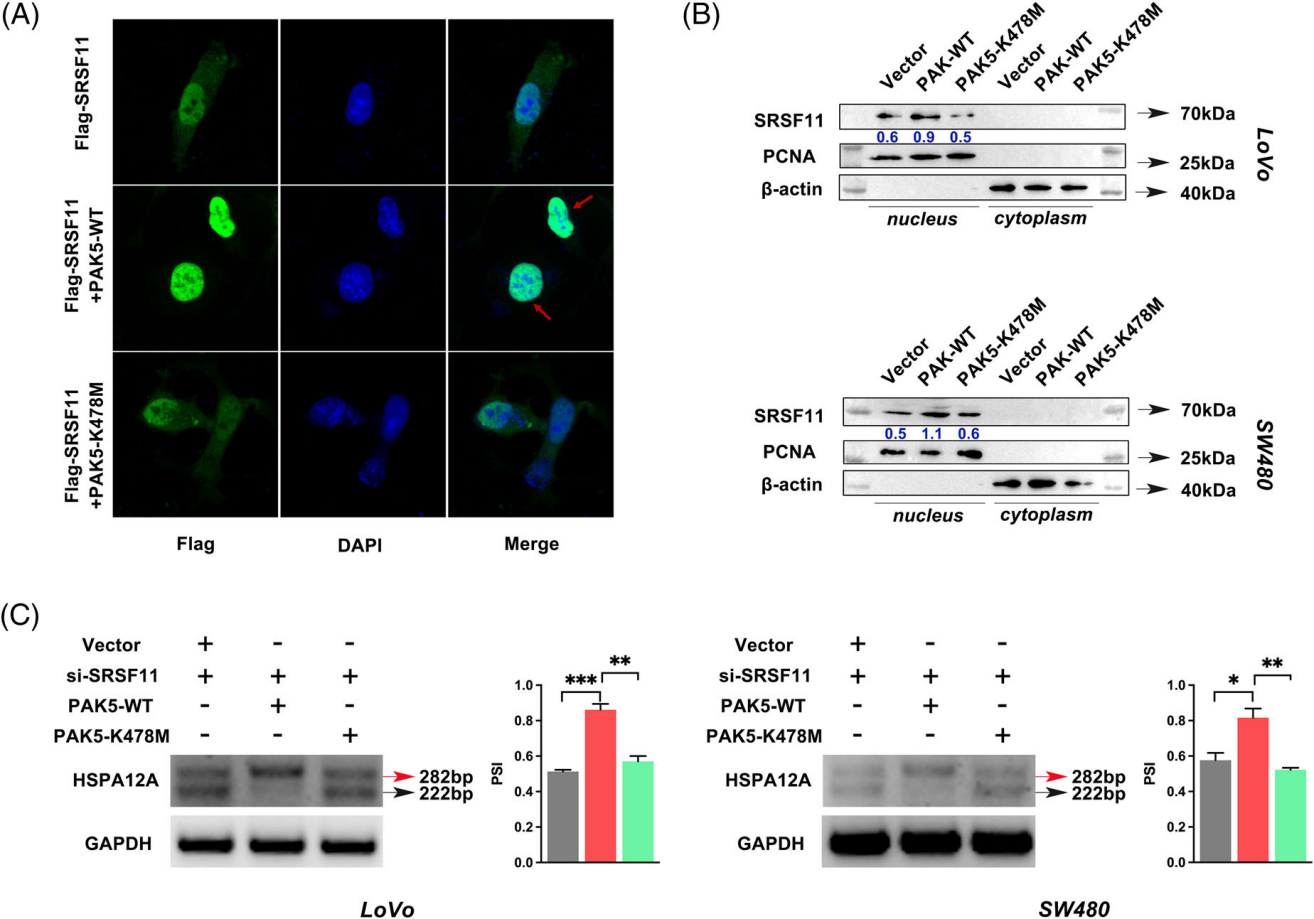
PAK5‐regulated phosphorylation facilitates the nuclear translocation of SRSF11 and inhibits the inclusion of HSPA12A exon 2. (A) Representative immunofluorescence images by confocal microscope indicate SRSF11 protein localization in LoVo cells. (B) WB analysis of SRSF11 protein levels in cytoplasm and nucleus, respectively. β‐actin and Lamin‐B1 were used as cytoplasmic and nuclear internal controls, respectively. (C) RT‐PCR results and quantification of HSPA12A RNA products measured as PSI after co‐transfection with indicated plasmid and siRNA in LoVo and SW480 cell lines (*N* = 3, SNK test; *p < .05, **p < .01, ***p < .001)

It has been observed that the phosphorylation status of serine/arginine‐rich proteins could affect their subcellular distribution and splicing activity. We wanted to find out how PAK5 affects SRSF11 subcellular localization based on our phosphorylation‐related interaction between PAK5 and SRSF11. As shown in Figure 7A, we co‐transfected Flag‐SRSF11 and MYC‐PAK5 or Vector plasmid into CRC cells and used a confocal fluorescence microscope to monitor the nucleocytoplasmic distribution of exogenous SRSF11 protein. The combined fluorescence indicated that PAK5 could increase SRSF11 nuclear translocation, whereas PAK5‐K478M overexpression had no effect on SRSF11 localization (Figure 7A). Further WB analysis validated the result that PAK5's phosphorylating regulation aided SRSF11 nuclear translocation (Figure 7B).

To determine the effect of PAK5‐regulated phosphorylation on SRSF11‐triggered AS, we co‐transfected si‐SRSF11 and PAK5 plasmids into CRC cells. As shown in Figure 7C, PAK5‐WT strongly increased HSPA12A AS, as expected, whereas co‐transfection of si‐SRSF11 and PAK5‐K478M had no effect. These findings suggested that PAK5 phosphorylation of SRSF11 enhanced its AS function on HSPA12A.

## 4 DISCUSSION

In this study, we investigated in‐depth SRSF11 and SRSF11‐triggered AS events, as well as upstream phosphorylation regulation in CRC. We discovered that SRSF11 induces critical splice‐switching of HSPA12A, which was linked to CRC metastasis in part through maintaining N‐cadherin RNA stability. SRSF11 was also demonstrated to be a substrate of PAK5 kinase and to be protected from Ub‐dependent degradation. These data suggest that SRSF11 promotes metastasis by activating tumour‐related AS events, emphasizing the importance of AS as a key regulator of metastasis.

Serine/arginine‐rich proteins, as members of the SF family, play a fundamental role in controlling AS events. So far, 12 members have been identified, with the 11th member, SRSF11, being discovered to be elevated with the most significant difference in CRC compared with normal adjacent tissues by our qRT‐PCR experiments (data not shown). SRSF11 has been reported to be implicated in the regulation of many AS events, but no comprehensive studies of its regulation in malignancies have been conducted.^38^ So far, only one clinical analysis has suggested that SRSF11 may operate as a prognosis‐related SF in ovarian carcinoma.^39^ Under certain conditions, SRSF11 cannot only stimulate but also prevent ES events. It has been reported to suppress the ES of F1γ pre‐mRNA and neuronal microexons while promoting tau, SMN2 and ZNF207 pre‐RNAs.^22^, ^23^, ^24^, ^25^, ^26^ Importantly, neither the clinical significance nor the biological function of SRSF11 in CRC are clarified. Our findings demonstrated that SRSF11 was substantially expressed in CRC and that its overexpression was associated with a worse prognosis. Further functional studies supported its pro‐metastatic role in CRC, as SRSF11 overexpression aided cell migration, invasion and wound healing ability in vitro, and promoted tumour metastasis in vivo.

Furthermore, we illustrated the SRSF11‐regulated AS landscape in CRC cell lines using RNA‐seq. SRSF11 expression affected 70 439 AS events, with ES being the most common splicing type (76%). One SF has been shown to perform antagonistic roles in activating or inhibiting AS processes in cancer cell lines.^40^ Our sequencing data also depicted this pattern within the context of SRSF11 regulation, demonstrating the complexities of AS control in various biological situations. It is worth noting that the proportion of ES patterns activated by SRSF11 was more than that of EI patterns because the upregulated PSI was significantly more than the downregulated PSI.

Canonical AS‐associated studies focused on the SF‐protein isoform axis. SETMAR pre‐mRNA, for example, was reported to be alternatively spliced to two protein isoforms in bladder cancer by NONO, leading to lymphatic metastasis.^41^ Besides, RBM4 pre‐mRNA was discovered to be translated into two distinct protein isoforms with opposite roles.^42^ Despite the fact that numerous researches have recently concentrated on the function of mRNA‐generating protein isoforms, the function of non‐coding RNAs (ncRNAs) produced by AS events has gradually been revealed, particularly in cancer.^43^, ^44^ However, the effects of ncRNA on tumour metastasis have received little attention. Based on RNA‐seq analysis and further CLIP and mini‐gene reporter assays, we filtered out HSPA12A pre‐RNA as the downstream AS target of SRSF11. And HSPA12A‐Ex2+ ncRNA phenocopied the pro‐metastatic activity of SRSF11 overexpression via Transwell and wound healing assays. EMT is a critical process that initiates tumour metastasis and is defined by the activation of a number of hallmarks, including N‐cadherin.^45^ In this study, we discovered that knocking down of HSPA12A‐Ex2+ significantly inhibited N‐cadherin expression; however, the underlying mechanism remains unknown. Nonetheless, we hypothesize that it is due to the role of the ncRNA HSPA12A‐Ex2+ in preserving N‐cadherin RNA stability, as ncRNA–mRNA interactions can improve the mRNA stability,^46^ and we demonstrated the probable binding of HSPA12A‐Ex2+ with N‐cadherin via an online tool. In brief, we hypothesized that the 60 bp length exon 2 of HSPA12A would be important for competing with endogenous N‐cadherin siRNAs and therefore sparing N‐cadherin mRNA from degradation. However, the concrete mechanism remains to be further investigated.

In addition, we applied a consensus analysis of the ES events regulated by SRSF11 using RNA‐seq data and found two peaks with overlapping sequences that matched a 6‐mer motif (GACC/TCA) in exon 2 of HSPA12A. Further mini‐gene reporter experiments with mutant plasmids confirmed the necessity of the 6‐mer motif for SRSF11 AS on HSPA12A. Certain serine/arginine‐rich proteins, including SRSF1, SRSF2, SRSF5 and SRSF6, have a preferred motif via which they exert the splicing processes.^47^, ^48^, ^49^, ^50^ Co‐incidentally, we found that our inferred preferential motif GACC/TCA of SRSF11 was similar to the preferential motif of SRSF2, implying a synergistic relationship between these two SFs.

The serine‐rich proteins piqued our interest in examining the phosphorylation regulation between SRSF11 and PAK5 kinase, the latter of which is a Ser/Thr protein kinase that has oncogenic functions in diverse types of cancer.^51^ Because the previously described SRPK, CLK and topoisomerase 1 families all tended to phosphorylate serine sequences,^19^ we concentrated on the serine‐rich RS domain. Given the lack of a specific antibody detecting the phosphorylated status of SRSF11, Phos‐tag SDS‐PAGE experiments were applied to differentiate the phosphorylated and unphosphorylated status of proteins by using a normal antibody.^52^ PAK5 phosphorylates SRSF11 at serine 287, as anticipated. The neighbouring AA of S287 is lysine (K288), and canonical Ub occurs on lysine residues. Conducting a structure prediction of SRSF11 protein through the AlphaFold Protein Structure Database (https://www.alphafold.ebi.ac.uk/entry/Q05519),^53^ we deduced that the phosphorylation of S287 affected the Ub of the adjacent K288. Further Ub‐IP experiments verified that PAK5 phosphorylation on SRSF11 on the S287 site was responsible for the suppression of SRSF11 Ub‐dependent degradation, reinforcing our hypothesis that S287 phosphorylation might interfere with the Ub of the nearby K288 site.

In conclusion, we discovered that upregulated SRSF11 and elevated exon 2‐inclusive HSPA12A were both related to enhanced metastatic ability and worse prognosis in CRC patients. Our findings demonstrate that SRSF11 can be phosphorylated by PAK5 and hence retains protein stability. Advanced research in different cancer types was required to determine whether modulating AS by targeting serine/arginine‐rich family members be widely employed in pan‐cancer. Overall, our findings implicate SRSF11 in mediating CRC metastasis by suppressing the ES of HSPA12A pre‐RNA (graphical abstract), suggesting that it might serve as both a potential therapeutic target and a prognostic indicator in CRC patients.

## FUNDING INFORMATION

National Natural Science Foundation of China, Grant/Award Number: 81872080

## CONFLICT OF INTEREST

The authors declare no potential conflicts of interest.

## Supporting information

Supporting Information

Supporting Information

Supporting Information

Supporting Information

Supporting Information

Supporting Information
