# Supplementary figures and images for: Alternative splicing of HSPA12A pre‐RNA by SRSF11 contributes to metastasis potential of colorectal cancer
Source: Clin Transl Med. 2022 Nov 17;12(11):e1113. doi: 10.1002/ctm2.1113 (PMC9670187; doi:10.1002/ctm2.1113)

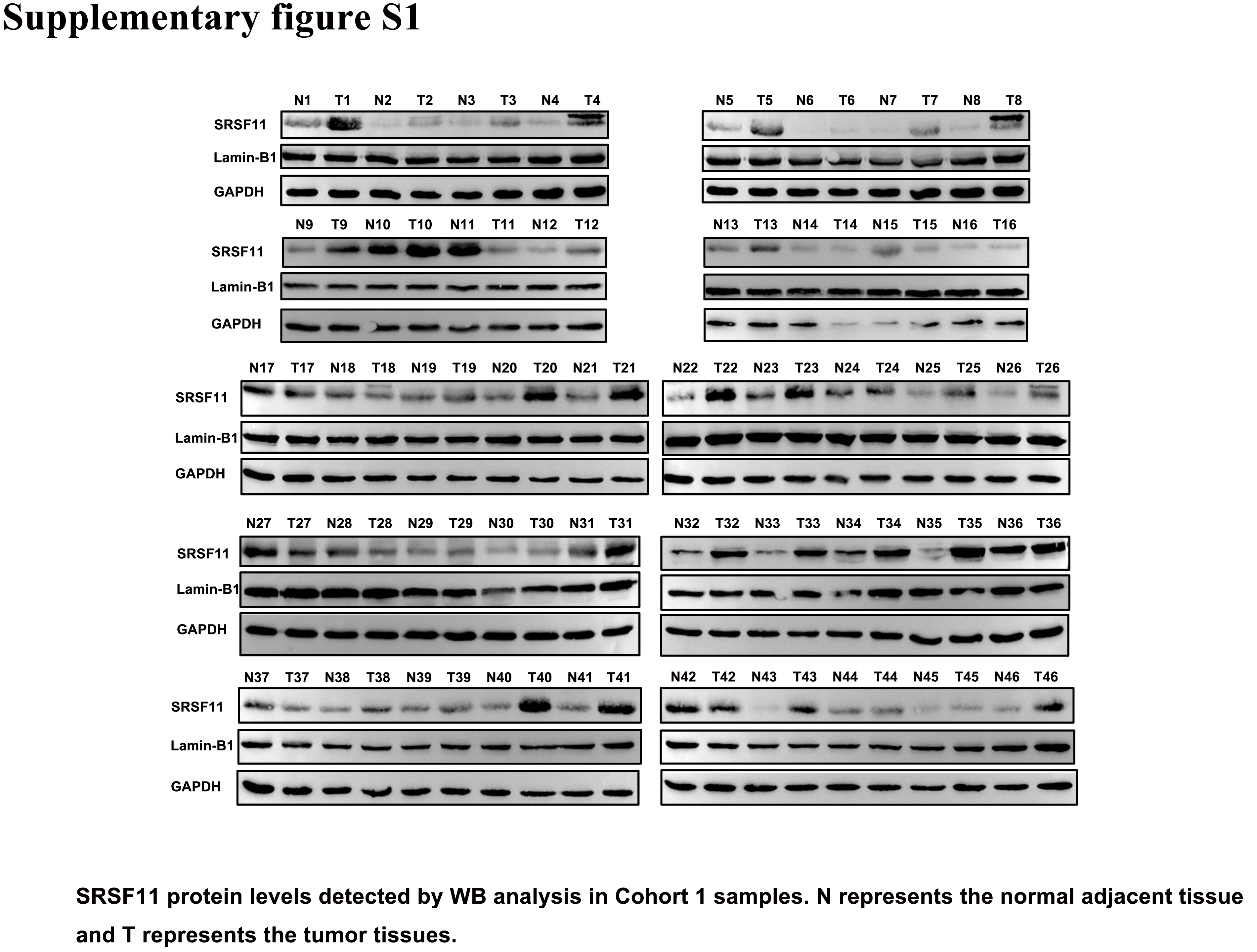

Supplement: Supplementary file 1 — Supporting Information [file CTM2-12-e1113-s004.tif]

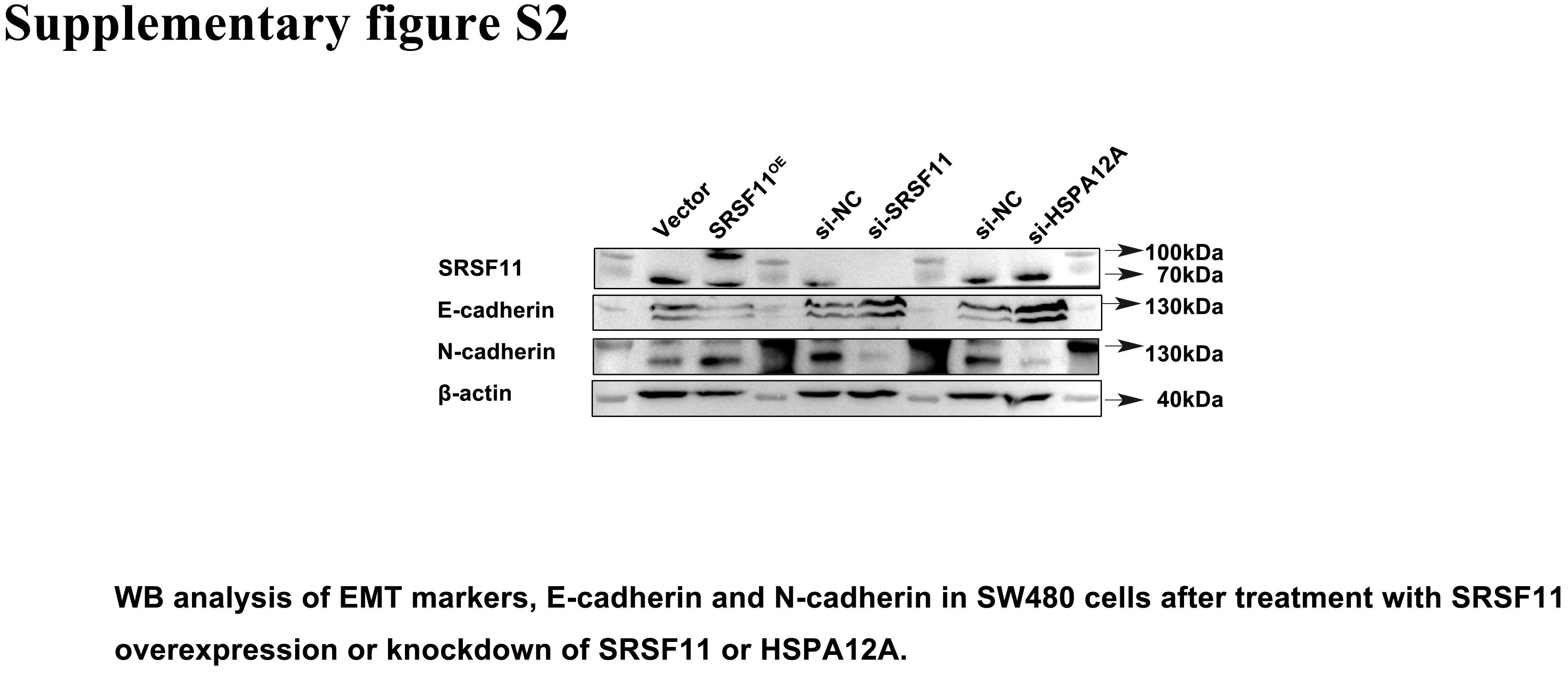

Supplement: Supplementary file 2 — Supporting Information [file CTM2-12-e1113-s002.tif]

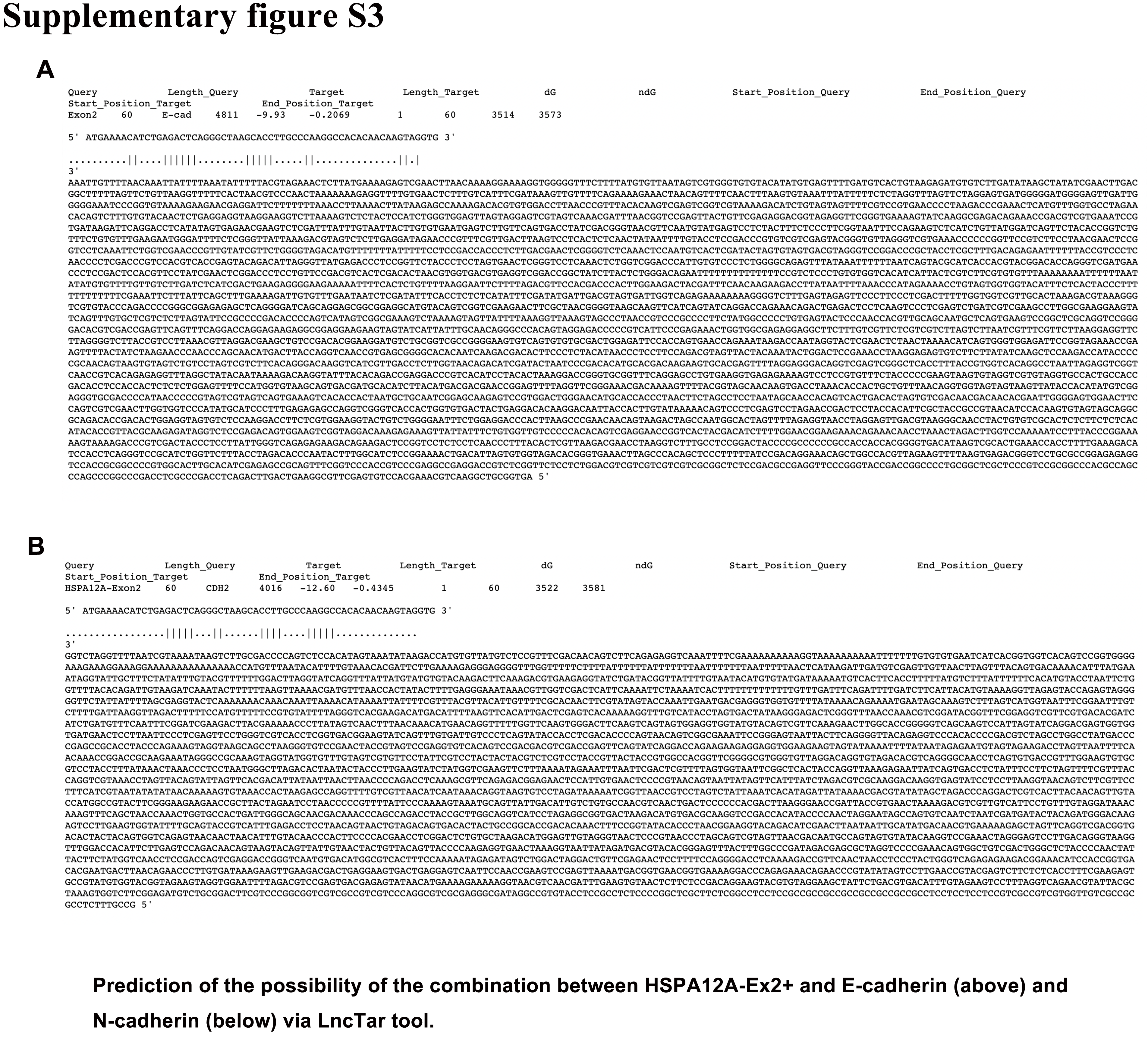

Supplement: Supplementary file 3 — Supporting Information [file CTM2-12-e1113-s006.tif]

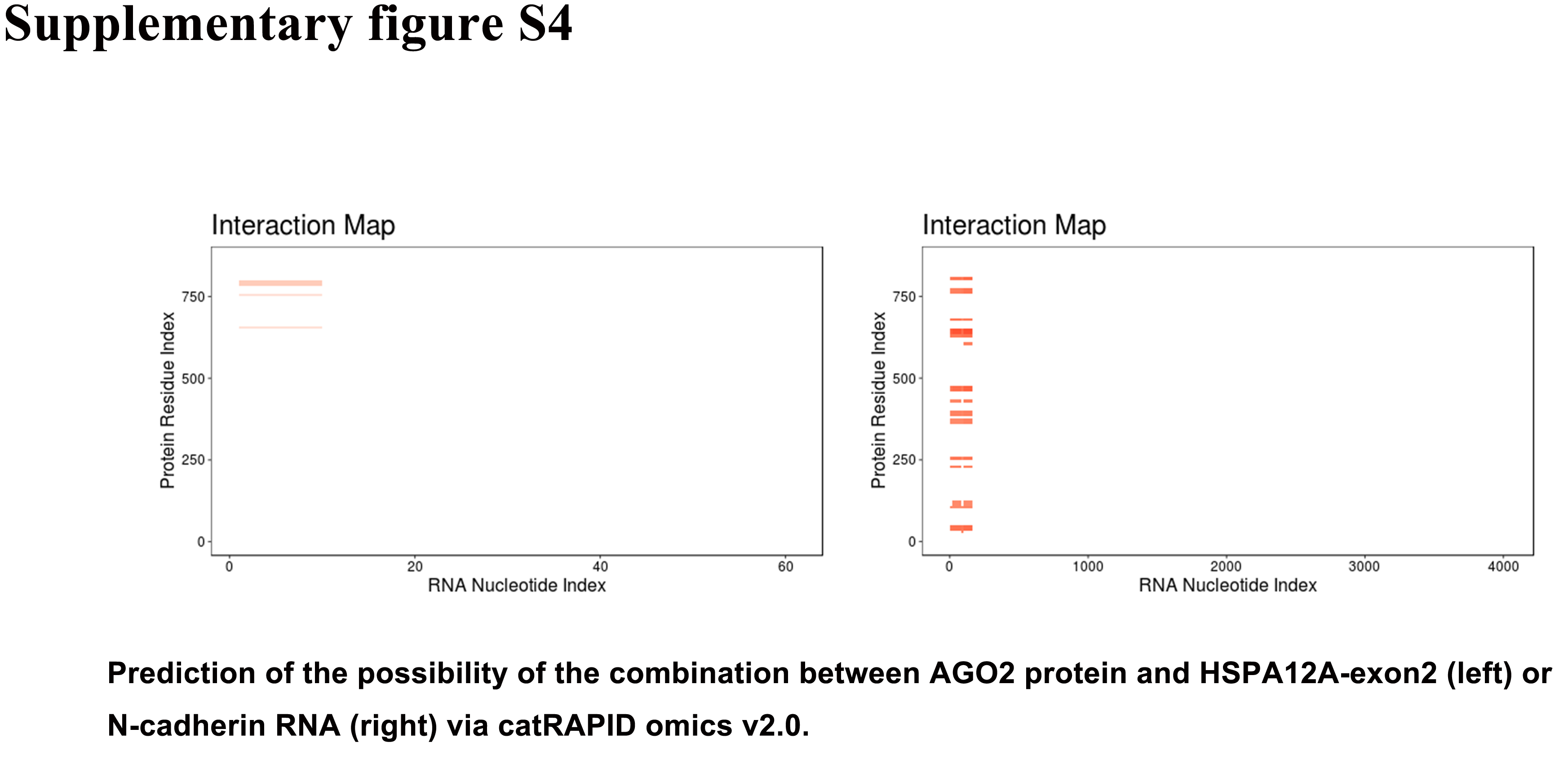

Supplement: Supplementary file 4 — Supporting Information [file CTM2-12-e1113-s003.tif]

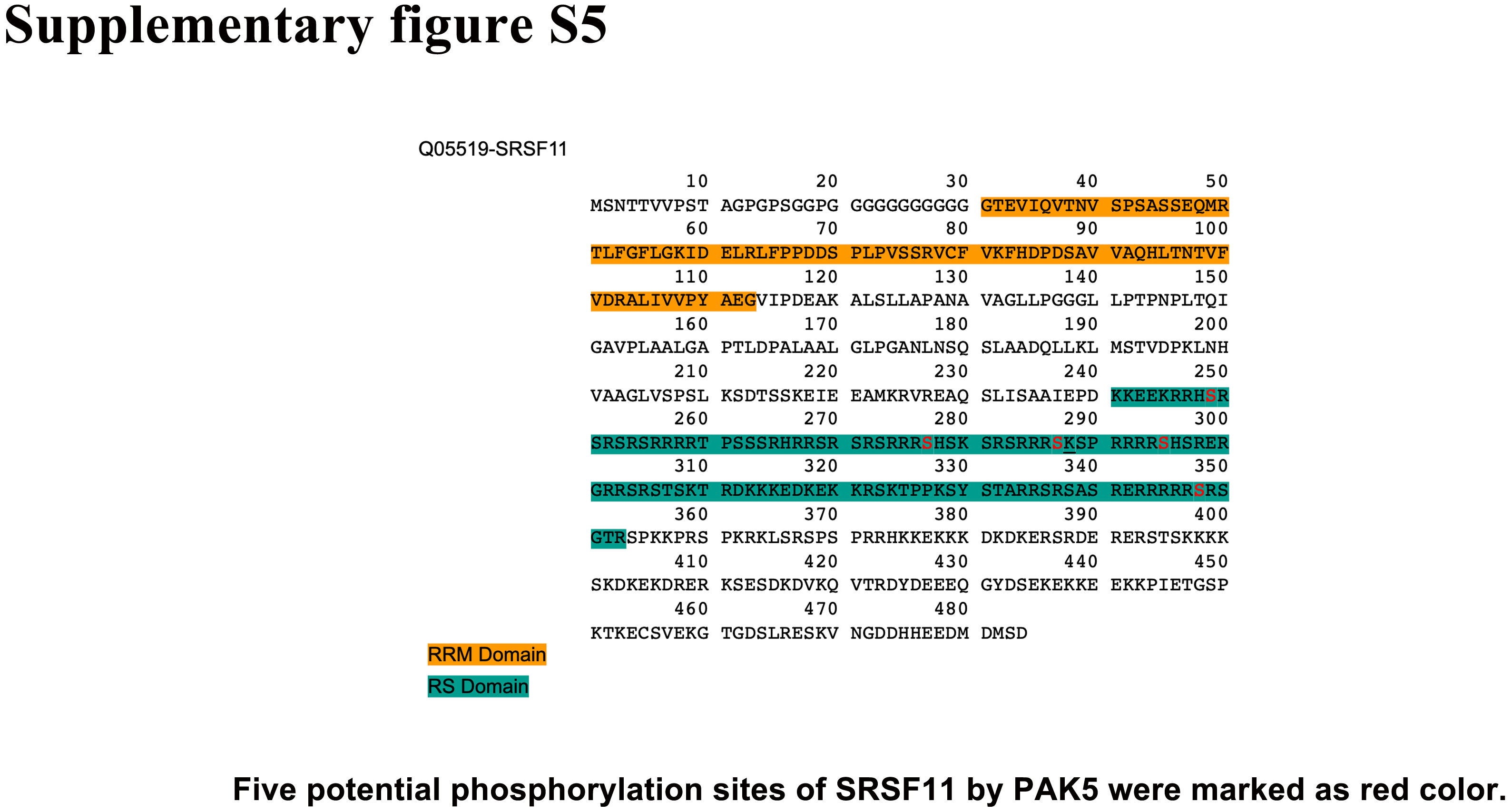

Supplement: Supplementary file 5 — Supporting Information [file CTM2-12-e1113-s001.tif]
