# Supplementary material for: Alternative splicing of HSPA12A pre‐RNA by SRSF11 contributes to metastasis potential of colorectal cancer
Source: Clin Transl Med. 2022 Nov 17;12(11):e1113. doi: 10.1002/ctm2.1113 (PMC9670187; doi:10.1002/ctm2.1113)
Supplement: Supplementary file 6 — Supporting Information [file CTM2-12-e1113-s005.docx]

**Supplementary Figures Captions**

**Supplementary Figures S1:** SRSF11 protein levels detected by WB analysis in Cohort 1 samples. N represents the normal adjacent tissue and T represents the tumor tissues.

**Supplementary Figures S2:** WB analysis of EMT markers, E-cadherin and N-cadherin in SW480 cells after treatment with SRSF11 overexpression or knockdown of SRSF11 or HSPA12A.

**Supplementary Figures S3:** Prediction of the possibility of the combination between HSPA12A-Ex2+ and E-cadherin (above) and N-cadherin (below) via LncTar tool.

**Supplementary Figures S4:** Prediction of the possibility of the combination between GO2 protein and HSPA12A-exon2 (left) or N-cadherin RNA (right) via catRAPID omics v2.0.

**Supplementary Figures S5:** Five potential phosphorylation sites of SRSF11 by PAK5 were marked as red color.
